# Xilei San Ameliorates Experimental Colitis in Rats by Selectively Degrading Proinflammatory Mediators and Promoting Mucosal Repair

**DOI:** 10.1155/2014/569587

**Published:** 2014-07-10

**Authors:** Yongbiao Hao, Kazuko Nagase, Kazutoshi Hori, Shenglan Wang, Yoko Kogure, Ken Fukunaga, Shinichiro Kashiwamura, Satoshi Yamamoto, Shiro Nakamura, Junxiang Li, Hiroto Miwa, Koichi Noguchi, Yi Dai

**Affiliations:** ^1^Department of Pharmacy, School of Pharmacy, Hyogo University of Health Sciences, 1-3-6 Minatojima, Chuo-ku, Kobe, Hyogo 650-8530, Japan; ^2^Division of Gastroenterology, Department of Internal Medicine, Hyogo College of Medicine, Nishinomiya, Hyogo 663-8501, Japan; ^3^Division of Internal Medicine, Department of Inflammatory Bowel Disease, Hyogo College of Medicine, Nishinomiya, Hyogo 663-8501, Japan; ^4^Traditional Medicine Research Center, Chinese Medicine Confucius Institute at Hyogo College of Medicine, Kobe, Hyogo 650-8530, Japan; ^5^Department of Anatomy and Neuroscience, Hyogo College of Medicine, Nishinomiya, Hyogo 663-8501, Japan; ^6^General Education Center, Hyogo University of Health Sciences, Kobe, Hyogo 650-8530, Japan; ^7^Department of Gastroenterology, Dongfang Hospital, Beijing University of Chinese Medicine, Beijing, 100078, China

## Abstract

Xilei san (XLS), a herbal preparation widely used in China for erosive and ulcerative diseases, has been shown to be effective in ulcerative colitis (UC). The present experiments were conducted to assess its efficacy and determine its mechanism of action in a rat model that resembles human UC. The model was induced by adding 4% dextran sulfate sodium (DSS) to the rats' drinking water for 7 days. XLS was administered daily by retention enema from day 2 to day 7; the rats were sacrificed on day 8. The colon tissues were obtained for further experiments. A histological damage score and the activity of tissue myeloperoxidase were used to evaluate the severity of the colitis. The colonic cytokine levels were detected in a suspension array, and epithelial proliferation was assessed using Ki-67 immunohistochemistry. Intrarectal administration of XLS attenuated the DSS-induced colitis, as evidenced by a reduction in both the histological damage score and myeloperoxidase activity. It also decreased the levels of proinflammatory cytokines, but increased the mucosal repair-related cytokines. In addition, the epithelial Ki-67 expression was upregulated by XLS. These results suggest that XLS attenuates DSS-induced colitis by degrading proinflammatory mediators and promoting mucosal repair. XLS could be a potential topical treatment for human UC.

## 1. Introduction

Inflammatory bowel disease (IBD) is characterized by chronic uncontrolled episodes of gastrointestinal inflammation [1]. Ulcerative colitis (UC), a major phenotype of IBD, is characterized by damage involving the mucosa and submucosa of the colon; bloody diarrhea is the major symptom. Inducing remission and preventing relapse are the primary goals in the management of UC. According to current consensus-based guidelines [2–4], the medical treatment for UC patients should take into consideration the clinical activity (mild, moderate, or severe) and the extent of colonic involvement (proctitis, left-sided colitis, or pancolitis). In patients with mild-to-moderate proctitis, mesalazine suppositories are often used as the first-line treatment. In a double-blind randomized study, we showed significant clinical and endoscopic efficacies of Xilei san (XLS) suppositories as well as their safety, in proctitis patients refractory to conventional topical therapy [5].

XLS is a compounded prescription of Chinese medicine, consisting of a fixed dose combination of the following well-characterized and standardized eight natural herbal or mineral substances: natural indigo (indigo naturalis), cow bezoar (calculus bovis), pearl powder (margarita), watermelon frost (mirabilitum praeparatum), calcitum (gypsum rubrum), borax (natrium biboricum), ammonium chloride (sal ammoniac), and borneol (borneolum syntheticum). In China, XLS is traditionally used in disorders that feature erosion or ulceration of mucosa or skin, including oral ulcer, esophagitis, erosive gastritis, peptic ulcer, chronic cervicitis, and UC [6–12]. Although a few studies have investigated the mechanism of action of XLS in an animal model [11], the exact pharmacological steps of XLS in UC have never been fully elucidated.

Clinical studies with herbal therapy in IBD have shown promising efficacy data and an acceptable safety profile; however, these remain limited and heterogeneous, and high-quality animal trials are still lacking [13]. With this background in mind, we investigated the pharmacological mechanism of the anti-inflammatory effects and mucosal healing of XLS on rats with dextran sulfate sodium- (DSS-) induced acute colitis—a condition that shares not only clinical and structural features with human UC but also pathophysiological and immunological characteristics [14–16].

## 2. Materials and Methods

### 2.1. Experimental Animals

All the animal experimental procedures previously approved by the Committee on Animal Research in Hyogo University of Health Sciences (number 2010-22) were performed in accordance with the guidelines on animal care of the National Institutes of Health. Adult male Sprague-Dawley (SD) rats (200–250 g; Japan SLC, Inc., Shizuoka, Japan) were housed in collective cages (3 rats per cage) at 24 ± 1°C under a 12 h-light/dark cycle, with free access to food and sterile tap water. All animals were allowed to adapt for 3 days before the experiments were begun. All experiments were performed during the light phase of the cycle.

### 2.2. DSS-Induced Colitis

DSS (MW 36–50 KDa, MP Biomedical, California, USA) was dissolved in sterilized tap water and presented to the rats at a final concentration of 4% w/v for 7 consecutive days. Fresh DSS solution was provided every day. Negative control healthy rats (noncolitis) received only sterilized tap water.

### 2.3. Treatment Protocol

The rats were randomly allocated to 4 groups: control rats treated with saline (water + saline); control rats treated with XLS (water + XLS); rats with DSS-induced colitis treated with saline (DSS + saline); and rats with DSS-induced colitis treated with XLS (DSS + XLS). XLS was prepared as a powder from a product commercially supplied by Beijing Tong Ren Tang Group Co., Ltd., Beijing, China. From day 1 to day 7, DSS solution was orally administered,* ad libitum*, to the DSS + saline and the DSS + XLS groups to induce colitis. Sterilized tap water was given to the rats in the other groups. On the following day, that is, day 2, the rats in the water + XLS and DSS + XLS groups were given XLS solution (0.3 g in 0.6 mL saline) intrarectally, while the remaining rats received only saline. After the rats were anesthetized intraperitoneally (i.p.) with pentobarbital sodium (50 mg/kg), the XLS solution was delivered to the colon at a depth of 8 cm from the anus, using a plastic circular needle. The retention enema, maintained for 2 h, was given daily from day 2 to day 7. After an overnight fast, the rats were killed on day 8, as described below. Body weight and colon length were measured at day 8.

### 2.4. Histological Analysis for Scoring Inflammation

The rats were deeply anesthetized with pentobarbital sodium (50 mg/kg, i.p.) and then perfused transcardially with 250 mL of 0.1 M phosphate buffer (pH 7.4) containing 1% paraformaldehyde (Nacalai Tesque, Kyoto, Japan) followed by 500 mL of the same buffer containing 4% paraformaldehyde. Colon lengths of 8 cm were removed and cut into 6 segments, each measuring 0.5 cm, starting from anus and cutting at 1 cm intervals. The sections were fixed in 4% buffered paraformaldehyde for 3 days at 4°C. The tissues were then transferred to a 30% solution of sucrose (w/v) for 1 day for cryoprotection and then sliced on a cryostat at 6 *μ*m thickness. The sections were mounted on silane-coated slides and stored at −80°C until used. After staining with hematoxylin and eosin (HE) (Wako Pure Chemical Industries, Ltd., Osaka, Japan), the degree of damage and inflammation was histologically assessed, and the histological damage score was calculated according to the following three parameters, which were scored on a scale of 0–4: (1) total lateral length of erosion, defined as loss of mucosal tissue (0, 0 mm; 1, 0.01–0.49 mm; 2, 0.50–0.99 mm; 3, 1.00–1.49 mm; 4, ≥1.50 mm); (2) total lateral length of inflammation, defined as a significantly excessive infiltration of leukocytes to the mucosa (0, 0 mm; 1, 0.01–0.99 mm; 2, 1.00–1.99 mm; 3, 2.00–2.99 mm; 4, ≥3.00 mm); and (3) a polymorphonuclear cell count of the densest mucosal area (high-power field, ×400), (0, 0–29; 1, 30–59; 2, 60–89; 3, 90–119; 4, ≥120). The degree of histological damage was then scored from 0 to 12.

### 2.5. Assay for Myeloperoxidase (MPO) Activity

Colonic MPO activity was assayed according to a method previously described [17]. Rats were sacrificed by decapitation. A 4 cm length of colon, measured from the anus, was removed and washed with phosphate-buffered saline over ice. The colonic mucosa was scraped off with a glass slide and then suspended in 1 mL of 50 mM potassium phosphate buffer (pH 6.0) containing 0.5% hexadecyl trimethyl ammonium bromide (Sigma Chemical Co., St. Louis, Missouri, USA). The suspension was next homogenized with an ultrasonic homogenizer and then centrifuged. MPO activity in the supernatant was assayed spectrophotometrically at 25°C by mixing 0.1 mL of the supernatant with 2.9 mL of 50 mM phosphate buffer (pH 6.0) containing 0.167 mg/mL* o*-dianisidine dihydrochloride (Sigma Chemical Co., St. Louis, Missouri, USA) and 0.0005% hydrogen peroxide. The change in absorbance at 460 nm was measured after 15 min with a spectrophotometer (Beckman Coulter, Inc., California, USA). The results are expressed as optical density (OD) per total protein of tissue.

### 2.6. Measurement of Cytokines and Chemokines

Rats were anesthetized with pentobarbital (50 mg/kg) and killed by decapitation. The 2 cm length colon was removed starting from anus, washed with wash buffer (Bio-Rad Laboratories, Inc., Hercules, California, USA), and homogenized in Bio-Plex cell lysis buffer (Bio-Rad Laboratories, Inc., Hercules, California, USA). After centrifugation, the supernatants were collected and stored at −80°C until further use. The supernatants were used to assay cytokines and chemokines using Bio-Plex Pro Rat Cytokine 24-Plex Assay kit (Bio-Rad Laboratories, Inc., Hercules, California, USA). The following cytokines and chemokines were quantified: interleukin- (IL-) 1*α*, IL-1*β*, IL-2, IL-4, IL-5, IL-6, IL-7, IL-10, IL-12, IL-13, IL-17, IL-18, erythropoietin (EPO), granulocyte-colony stimulating factor (G-CSF), granulocyte macrophage-colony stimulating factor (GM-CSF), growth-related oncogene/keratinocyte-derived chemokine (GRO/KC), interferon-*γ* (IFN-*γ*), macrophage colony-stimulating factor (M-CSF), monocyte inflammatory protein-1*α* (MIP-1*α*), MIP-3*α*, regulated on activation normal T cell expressed and secreted (RANTES), tumor necrosis factor (TNF)-*α*, vascular endothelial growth factor (VEGF), and monocyte chemoattractant protein (MCP-1).

### 2.7. Ki-67 Immunohistochemistry

Frozen sections (6 *μ*m) of colon were made, as described above, and processed for Ki-67 immunohistochemistry. The procedure was described in detail in a previous study [18]. A 1 : 500 solution of the primary monoclonal antibody of Ki-67 (clone MM1; Novocastra Laboratories, Newcastle, UK) was used. After reacting with diaminobenzidine, the sections were counterstained with hematoxylin, washed in Milli-Q water (Millipore Corporation, Billerica, MA, USA), air-dried, and dehydrated via an alcohol gradient (70, 80, 90, 95, and 100%). After the alcohol was replaced by xylene, the sections were coverslipped. The labeling index (LI) was calculated according to a previously described method [19] and expressed as a percentage of the positive cells among 1000 cells. To remove an observation bias, epithelial cells on tubules, which had a U-shaped configuration to enable a fair observation from the surface to the basal side, were counted close to the erosive or inflamed area.

### 2.8. Statistical Analysis

All data are reported as mean ± standard error (SE). The Steel-Dwass test was used for nonparametric all-pairs multiple comparison in each group of data. Differences of *P* < 0.05 or less were considered to be significant. The statistical tests were performed using JMP Software version 10.0 (SAS Institute Inc., Cary, North Carolina, USA).

## 3. Results

### 3.1. Body Weight and Colon Length

Body weight was significantly loosed in the DSS group compared with the control group (DSS group versus water + saline group: 245.8 ± 10.5 g [*N* = 11] versus 268.5 ± 17.7 g [*N* = 10], *P* < 0.05). Likewise, in the DSS group, colon length (cm) was significantly shorter compared with the water + saline group (DSS group versus water + saline group: 16.8 ± 1.2 cm [*N* = 11] versus 19.2 ± 1.6 cm [*N* = 10], *P* < 0.05). However, quantification of the body weight or the colon length did not demonstrate significant alteration between DSS group and DSS + XLS group (body weight: DSS group versus DSS + XLS group: 245.8 ± 10.5 g [*N* = 11] versus 231.2 ± 21.8 g [*N* = 12], *P* = 0.13, colon length: DSS group versus DSS + XLS group: 16.8 ± 1.2 cm [*N* = 11] versus 17.0 ± 0.8 cm [*N* = 12], *P* = 0.90).

### 3.2. Histological Features

**Figure 1:**
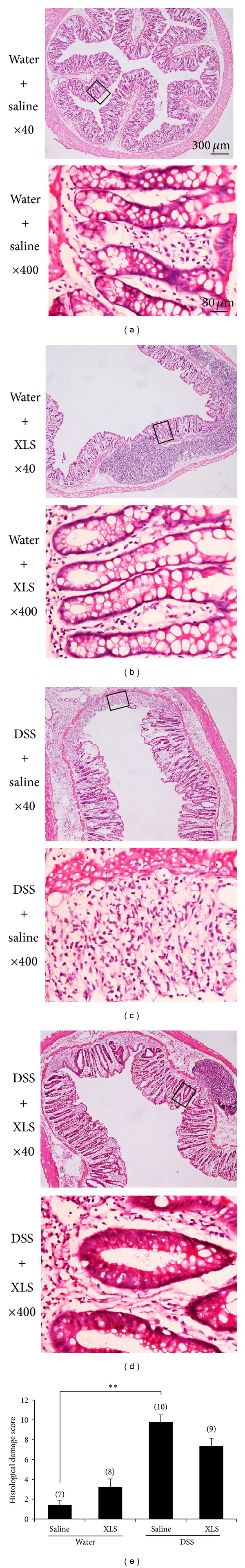
XLS attenuated DSS-induced colonic mucosal damage in rats. (a) Water + saline (sterile tap water and saline); (b) water + XLS (sterile tap water and 0.3 g XLS); (c) DSS + saline (4% DSS and saline); (d) DSS + XLS (4% DSS and 0.3 g XLS) (HE staining, ×40 and ×400 original magnification); and (e) total histological damage score. Numbers in brackets represent the sample numbers of each group. **P < 0.01 versus water + saline.

Representative histological images of HE-stained colon sections from each group are shown in Figure 1. In contrast to that of the control group (Figure 1(a)), the histology of tissues from the DSS-induced colitis revealed erosions and a remarkable infiltration of inflammatory cells with some increase of fibroblasts (Figure 1(c)). In the DSS + XLS group, after intrarectal administration of XLS, crypt regeneration and restoration with mild infiltration of inflammatory cells were observed (Figure 1(d)). Interestingly, the water + XLS group showed a significant accumulation of Peyer's patches in the submucosa (Figure 1(b)). As shown in the bar graph (Figure 1(e)), the histological damage score increased significantly more in the DSS + saline group than in the water + saline group (9.80 ± 0.70 [*N* = 10] versus 1.43 ± 0.48 [*N* = 7], *P* < 0.01). Treatment with XLS tended to protect against the histological damage induced by DSS, when compared to the DSS + saline group (7.33 ± 0.82 [*N* = 9] versus 9.80 ± 0.70 [*N* = 10], *P* = 0.18, not statistically significant).

### 3.3. Decrease in Colonic MPO Activity in DSS-Treated Rats due to XLS

**Figure 2:**
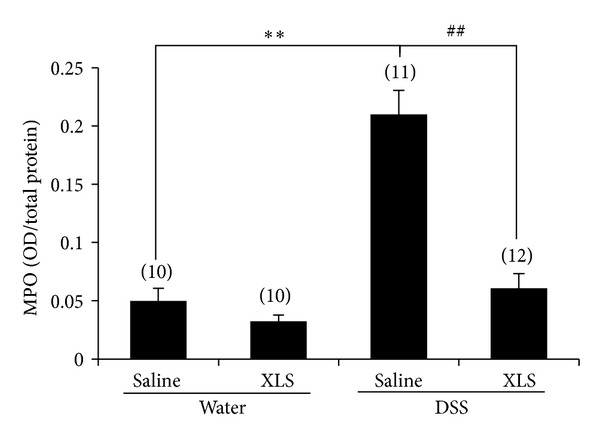
XLS reduced colonic MPO activity in DSS-treated rats. Numbers in brackets represent the sample numbers of each group. **P < 0.01 versus water + saline, ^##^ *P* < 0.01 versus DSS + saline.

As expected, the MPO activity in colonic mucosa was significantly greater in the DSS + saline group than in the water + saline group (0.21 ± 0.02 OD/total protein [*N* = 11] versus 0.05 ± 0.01 [*N* = 10], *P* < 0.01). This difference was significantly less when the group given DSS + XLS was compared to the one given DSS + saline (0.06 ± 0.01 OD/total protein [*N* = 12] versus 0.21 ± 0.02 [*N* = 11], *P* < 0.01) (Figure 2). These results suggest that XLS inhibits granulocytic infiltration in DSS-induced colitis.

### 3.4. XLS Suppression of the Increase in Inflammatory Cytokines and Chemokines Induced by DSS

**Figure 3:**
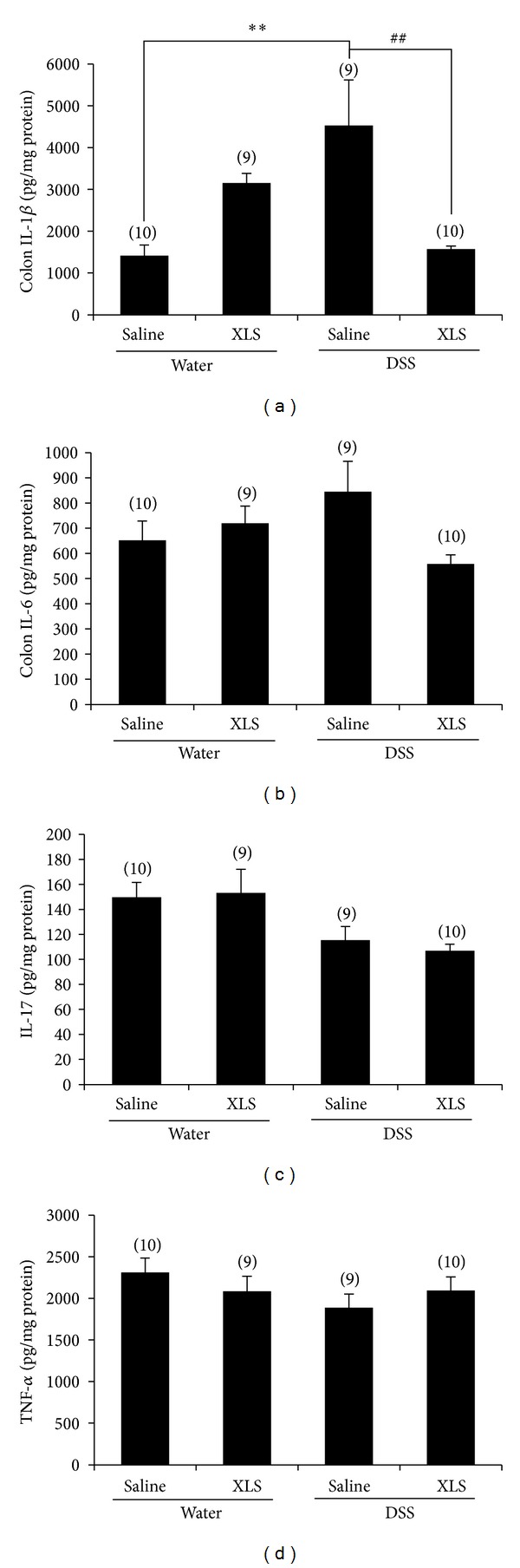
In rats, XLS suppressed the increase of a colonic cytokine of IL-1*β* (a) induced by DSS. IL-6 (b), IL-17 (c), and TNF-*α* (d) remained constant in DSS group compared with water group, which was comparable to that of XLS groups. Numbers in brackets represent the sample numbers of each group. **P < 0.01 versus water + saline, ^##^ *P* < 0.01 versus DSS + saline.

Proinflammatory cytokine IL-1*β*, which is secreted mainly by macrophages, may contribute to the tissue damage seen in DSS-induced colitis. The concentration of IL-1*β* in colonic tissue was significantly greater in the DSS + saline group than that in the water + saline group (4528.05 ± 1089.04 pg/mg protein [*N* = 9] versus 1413.61 ± 256.04 [*N* = 10], *P* < 0.01). A comparison of the IL-1*β* induced by DSS with and without XLS treatment shows that XLS prevents the elevation of IL-1*β*, as evidenced by the values for IL-1*β* in the DSS + XLS and the DSS + saline groups (1571.09 ± 70.72 pg/mg protein [*N* = 10] versus 4528.05 ± 1089.04 [*N* = 9], *P* < 0.01) (Figure 3(a)). The concentration of other proinflammatory cytokines, such as IL-6, IL-17, and TNF-*α*, was not significantly elevated in the DSS + saline group compared with water + saline group (847.28 ± 118.13 pg/mg protein [*N* = 9] versus 654.19 ± 74.44 [*N* = 10], *P* = 0.64 for IL-6; 115.37 ± 10.93 pg/mg protein [*N* = 9] versus 149.73 ± 11.87 [*N* = 10], *P* = 0.34 for IL-17, and 1888.52 ± 162.45 pg/mg protein [*N* = 9] versus 2313.05 ± 171.16 [*N* = 10], *P* = 0.43 for TNF-*α*, resp.) (Figures 3(b)–3(d)).

**Figure 4:**
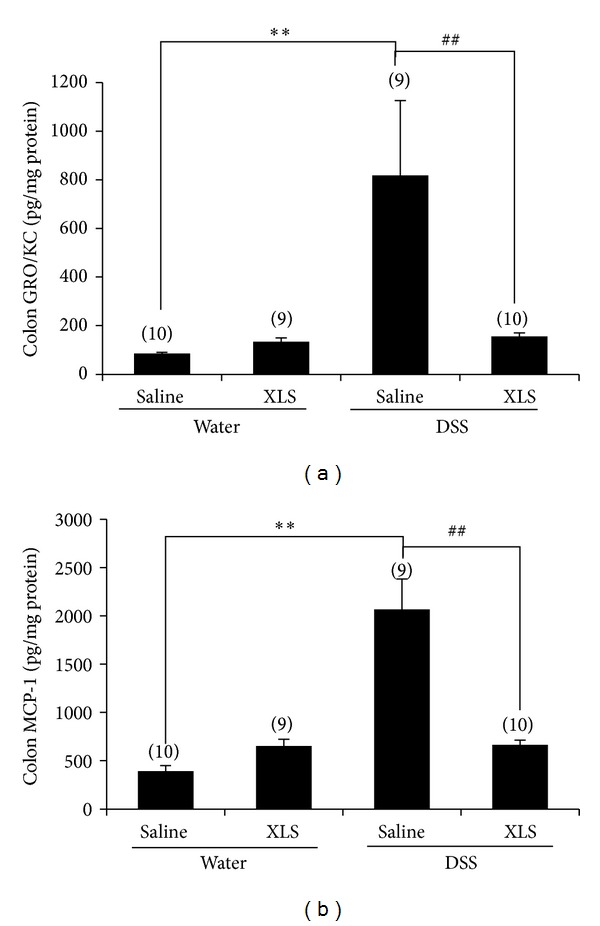
In rats, XLS inhibited the increase of the colonic chemokines (a) GRO/KC and (b) MCP-1 induced by DSS. Numbers in brackets represent the sample numbers of each group. **P < 0.01 versus water + saline, ^##^ *P* < 0.01 versus DSS + Saline.

In addition to the differences in the concentration of a proinflammatory cytokine IL-1*β*, two colonic chemokines—GRO/KC (Figure 4(a)) and MCP-1 (Figure 4(b))—were significantly greater in the DSS + saline group than in the water + saline group (817.92 ± 308.07 pg/mg protein [*N* = 9] versus 84.99 ± 4.73 [*N* = 10], *P* < 0.01 for GRO/KC, and 2065.03 ± 316.12 pg/mg protein [*N* = 9] versus 391.63 ± 59.02 [*N* = 10], *P* < 0.01 for MCP-1, resp.). Both GRO/KC and MCP-1 protein levels were suppressed by XLS, as seen when comparing the two groups, DSS + XLS, and DSS + saline (155.31 ± 15.38 pg/mg protein [*N* = 10] versus 817.92 ± 308.07 [*N* = 9], *P* < 0.01 for GRO/KC, and 666.11 ± 45.73 pg/mg protein [*N* = 10] versus 2065.03 ± 316.12 [*N* = 9], *P* < 0.01 for MCP-1, resp.).

### 3.5. XLS Upregulated Colonic Mucosal Repair-Related Cytokine Expression and Spurred Enterocyte Proliferation in DSS-Treated Rats

**Figure 5:**
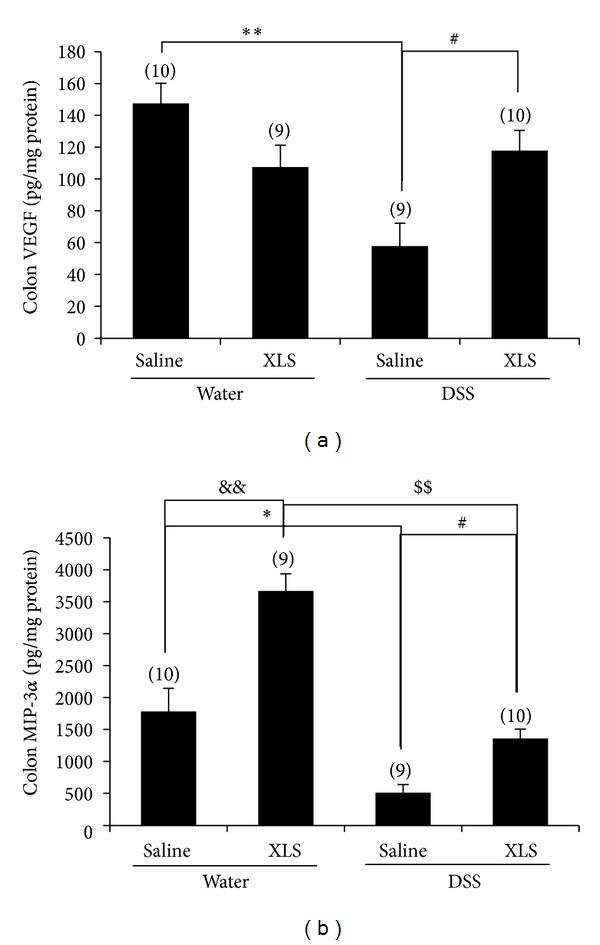
In rats, XLS upregulated colonic mucosal repair-related cytokine expression of (a) VEGF and (b) MIP-3*α*. Numbers in brackets represent sample numbers of each group. **P < 0.01 versus water + saline, *P < 0.05 versus water + saline, ^#^ *P* < 0.05 versus DSS + saline, ^&&^ *P* < 0.01 versus water + saline, and ^$$^ *P* < 0.01 versus DSS + XLS.

VEGF is a cytokine that promotes angiogenesis and MIP-3*α* functions in epithelial migration and mucosal barrier repair. Treatment with DSS reduced colonic VEGF (Figure 5(a)) and MIP-3*α* (Figure 5(b)) levels, as seen in the comparison between the assays for these moieties in the DSS + saline and water + saline groups (57.95 ± 14.20 pg/mg protein [*N* = 9] versus 147.42 ± 12.66 [*N* = 10], *P* < 0.01 for VEGF, and 511.64 ± 124.53 pg/mg protein [*N* = 9] versus 1781.36 ± 364.39 [*N* = 10], *P* < 0.05 for MIP-3*α* resp.), while XLS treatment significantly prevented that degradation, as evidenced by comparing the values obtained from the DSS + XLS and DSS + saline groups (117.75 ± 12.71 pg/mg protein [*N* = 10] versus 57.95 ± 14.20 [*N* = 9], *P* < 0.05 for VEGF, and 1358.20 ± 147.28 pg/mg protein [*N* = 10] versus 511.64 ± 124.53 [*N* = 9], *P* < 0.05 for MIP-3*α*, resp.). Furthermore, XLS treatment upregulated the MIP-3*α* expression in normal rats, as seen in the comparison of the MIP-3*α* from the water + XLS and the water + saline groups (3668.15 ± 265.42 pg/mg protein [*N* = 9] versus 1781.36 ± 364.39 [*N* = 10], *P* < 0.01) (Figure 5(b)).

**Figure 6:**
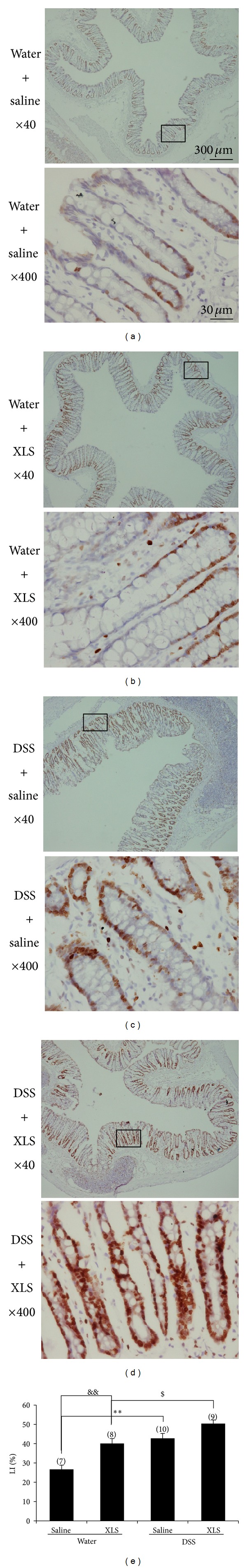
XLS stimulated enterocyte proliferation in DSS-treated rats. (a) Water + saline; (b) water + XLS; (c) DSS + saline; and (d) DSS + XLS (immunohistochemistry, ×40 and ×400 original magnification). (e) Cell proliferation was evaluated by LI, representative of the immunoreactivity of Ki-67 in colon sections. Numbers in brackets represent sample numbers of each group. **P < 0.01 versus water + saline, ^&&^ *P* < 0.01 versus water + saline, and ^$^ *P* < 0.05 versus DSS + XLS.

Ki-67, a nuclear protein expressed in all active phases of the cell cycle, is necessary for cell proliferation. To explore whether or not XLS could interfere with enterocyte proliferation, we evaluated the Ki-67 expression in colonic epithelium as a function of the LI. Ki-67 immunohistochemistry exhibited low expression in epithelial cells in the water + saline group (Figure 6(a)). In contrast, DSS treatment increased the Ki-67 expression as seen by comparing the water + saline and the DSS + saline groups (Figure 6(c)) (27 ± 2% in LI [*N* = 7] versus 43 ± 3% [*N* = 10], *P* < 0.01 in Figure 6(e)). Interestingly, XLS, as well, promoted the expression of Ki-67, as evidenced by the comparison between the water + saline and water + XLS groups (Figure 6(b)) (27 ± 2% in LI [*N* = 7] versus 40 ± 3% [*N* = 8], *P* < 0.01 in Figure 6(e)). The expression in the DSS + XLS group was even greater than that in the DSS + saline group (Figure 6(d)) (43 ± 3% in LI [*N* = 10] versus 50 ± 2% [*N* = 9], *P* = 0.19), although the difference was not statistically significant (Figure 6(e)). These data indicate that XLS stimulates enterocyte proliferation in DSS-induced colitis (Figure 6(d)).

## 4. Discussion

In the present study, we demonstrated that intrarectal administration of XLS suppresses DSS-induced colitis by decreasing the infiltration and migration of inflammatory cells into the colon, reducing colonic MPO activity, degrading the proinflammatory cytokines and chemokines, and promoting mucosal repair.

### 4.1. XLS Reduction of Local Infiltration, Migration of Inflammatory Cells, and Production of Inflammatory Cytokines

Inflammation of the intestinal mucosa is characterized by an excessive infiltration of such inflammatory cells as neutrophils and macrophages, which is accompanied by the production of proinflammatory cytokines [20]. Neutrophils play a crucial role in mediating tissue injury and clinical symptoms in colitis [21–23]. In the histological analysis, we found that neutrophilic infiltration was increased in the DSS rat colonic tissue. XLS treatment reduced this infiltration (Figure 1(d)). Neutrophilic infiltration correlates with tissue MPO activity, which represents the severity of DSS-induced colitis [24]. Although MPO is an enzyme that catalyzes the formation of hypochlorous acid, it also possesses cytokine-like properties and can activate neutrophils, with a resulting release of a wide range of inflammatory mediators [25]. In accord with this evidence, MPO activity was shown to significantly increase in rat colonic tissue with DSS-induced colitis, and the XLS treatment prevented this increase (Figure 2), a finding indicative of a reduction in neutrophilic infiltration and a decrease in colon damage.

Activated inflammatory cells may upregulate the production of cytokines in colon tissue, and cytokines then create a positive feedback loop that exacerbates the colonic damage [26]. Circulating IL-1*β*, IL-6, IL-17, and TNF-*α* play a key role in the pathogenesis of IBD [27]. Since IL-1*β* is involved in the early stage of DSS-induced colitis, the downregulation might be available for the treatment of patients with UC [28]. We found that XLS treatment significantly reduced IL-1*β* production in DSS-induced colitis, a finding that could indicate that XLS prevents neutrophil and macrophage infiltration. It is reported that IL-6 levels correlate with IBD activity [29]. Although upward tendency of IL-6 level was found in the DSS group, there was no significant difference compared with water group (Figure 3(b)). This may indicate a mild severity of inflammation in the present DSS model. On the other hand, IL-17 is a delayed-type immune reaction cytokine produced by Th17 and by CD8+ T cells during chronic inflammation [29, 30]. DSS colitis represents an acute model of colitis [30]; this idea supports that we could not detect significant changes of IL-17 expression on the present DSS colitis. In the present study, we evaluate the proinflammatory cytokines at 7 days after DSS treatment, the time point which may represent the early stage of the colitis. DSS may activate local and infiltrated macrophage to produce IL-1*β* prior to upregulation of other proinflammatory cytokines in this time point. Regarding these points of view, the colitis model in the present study may represent the early stage and/or mild severity of inflammation. Further studies are warranted to investigate the effect of XLS on these proinflammatory cytokines, such as IL-6, IL-17, and TNF-*α* in colitis.

The inflammatory cells in DSS-induced colitis may be chemically attracted to the intestinal mucosa by chemokines. In the present study, we observed that GRO/KC and MCP-1 were upregulated by DSS, while XLS significantly prevented the production of both GRO/KC and MCP-1 in DSS-induced colitis (Figure 4). Given that GRO/KC is a chemokine for neutrophils [31], while MCP-1 plays a chemoattractant role for macrophages [32], these findings suggest that XLS can reduce the infiltration of neutrophils and macrophages to the inflamed foci. Therefore, the XLS treatment may contribute to a decrease in cell migration by diminishing the production of chemotactic factors.

### 4.2. XLS Promotion of Epithelial Repair in the Damaged Colon

Intestinal mucosa has a zone of rapidly proliferating epithelial cells, which sustains injuries in response to stresses ranging from physiological daily digestive trauma to severe insults associated with ischemia, chemical insult, and infection. Mucosal repair in the acute phase of colitis is a complex process and often includes villus contraction, epithelial migration, and closure of the epithelial cell gap and tight junctions [33]. These processes require cytokines such as VEGF and MIP-3*α*. VEGF plays a pivotal role in the reconstruction of vascular cells [34], while MIP-3*α* contributes to efficient epithelial migration and mucosal barrier repair [35]. In the present study, we found that the expressions of VEGF and MIP-3*α* were markedly downregulated in DSS-induced colitis, while XLS treatment significantly inhibited the downregulation of these cytokines (Figure 5). These results indicate that XLS promotes epithelial repair. In addition to this observation, XLS treatment notably increased Ki-67 protein expression in colonic epithelium, with or without DSS-induced colitis. Ki-67, a nuclear protein associated with cellular proliferation, is present during all active phases of the cell cycle (G1, S, G2, and mitosis) but is absent from resting cells (G0) [36]. Therefore, the pathway underlying the XLS protection against colonic damage could be associated with proliferating enterocytes. Taken as a whole, these data indicate that XLS would be effective in promoting epithelial migration and mucosal barrier repair.

It was noteworthy that XLS treatment increased the expression of colonic MIP-3*α* (Figure 5(b)). In connection with the finding, we observed that lymphocytes accumulated in the submucosa (also known as Peyer's patch) in the XLS-treated colon (Figure 1(b)). MIP-3*α* is strongly chemotactic for lymphocytes and thus implicated in the formation and function of lymphoid tissue via the chemoattraction of lymphocytes and dendritic cells toward the epithelial cells around lymphoid tissue [37]. This accumulation of lymphocytes may participate in mucosal restoration by releasing mucosal repair-related cytokines, but determining the exact mechanism will require further investigation.

In this study, we assessed only the efficacy of the action of XLS on DSS-induced colitis in the acute phase [15]. Therefore, whether XLS exerts its action in the chronic phase by the same mechanism is still unclear. In addition, although we confirmed the efficacy of topical XLS administration on DSS-induced colitis, we need to investigate further whether or not systemic administration of the compound would also be effective. Moreover, because XLS is a mixture of Chinese medical herbs that include eight natural herbal or mineral substances, we cannot know without further investigation whether any one component or one substance in one component plays a special role in the healing process.

## 5. Conclusions

Our results show that topical treatment with XLS can ameliorate colitis. The action is associated with two possible mechanisms. First, XLS could prevent the release in colonic tissue of proinflammatory mediators, by inhibiting the influx of leukocytes into inflamed foci. Second, XLS may promote both mucosal repair and epithelial cell proliferation, which contribute to the mucosal restoration. Considering the evidence from our previous clinical reports [5, 38], we suggest XLS as a candidate for the topical treatment of UC.
